# Early CD4+ T Cell Responses Are Associated with Subsequent CD8+ T Cell Responses to an rAd5-Based Prophylactic Prime-Boost HIV Vaccine Strategy

**DOI:** 10.1371/journal.pone.0152952

**Published:** 2016-04-28

**Authors:** Edouard Lhomme, Laura Richert, Zoe Moodie, Chloé Pasin, Spyros A. Kalams, Cecilia Morgan, Steve Self, Stephen C. De Rosa, Rodolphe Thiébaut

**Affiliations:** 1 INSERM, ISPED, Centre INSERM U897-Epidemiologie-Biostatistique, Bordeaux, France; 2 Université Bordeaux, ISPED, Centre INSERM U897-Epidemiologie-Biostatistique, Bordeaux, France; 3 CHU de Bordeaux, Pôle de santé publique, Bordeaux, France; 4 INRIA SISTM, Talence, France; 5 Vaccine Research Institute (VRI), Créteil, France; 6 Vaccine and Infectious Disease Division, Fred Hutchinson Cancer Research Center, Seattle, Washington, 98109, United States of America; 7 HIV Vaccine Trials Network, Seattle, Washington, 98109, United States of America; 8 Infectious Diseases Unit, Department of Medicine, Vanderbilt University School of Medicine, Nashville, Tennessee, 37232, United States of America; Rush University, UNITED STATES

## Abstract

**Introduction:**

Initial evaluation of a candidate vaccine against HIV includes an assessment of the vaccine’s ability to generate immune responses. However, the dynamics of vaccine-induced immune responses are unclear. We hypothesized that the IFN-γ producing cytotoxic CD8+ (CD8+ IFN-γ+) T cell responses could be predicted by early IL-2 producing CD4+ (CD4+ IL-2+) helper T cell responses, and we evaluated this hypothesis using data from a phase I/II prophylactic HIV vaccine trial. The objective was to assess the dynamics and correlations between CD4+ IL-2+ T cell and CD8+ IFN-γ+ T cell responses after vaccination with a recombinant adenoviral serotype 5 (rAd5) HIV vaccine.

**Methods:**

We analyzed data from the HVTN 068 HIV vaccine trial, which evaluated the immunogenicity of two different strategies for prime and boost vaccination (rAd5-rAd5 vaccine versus DNA-rAd5) in 66 healthy volunteers. Spearman correlations between immunogenicity markers across time-points were calculated. CD8+ IFN-γ+ T cell response in the rAd5-rAd5 arm was modeled as a function of CD4+ IL-2+ T cell response and time using mixed effects regression models.

**Results:**

Moderate to high correlations (r = 0.48–0.76) were observed in the rAd5-rAd5 arm between the CD4+ IL-2+ T cell response at week 2 and later CD8+ IFN-γ+ T cell responses (weeks 2–52). Regression models confirmed this relationship with a significant association between the two markers: for a 1.0% increase in CD4+ IL-2+ T cells at week 2 post-prime, a 0.3% increase in CD8+ IFN-γ+ T cell responses across subsequent time points, including post-boost time points, was observed (p<0.01).

**Conclusion:**

These results suggest an early and leading role of CD4+ T cells in the cellular response to the rAd5-rAd5 vaccine and in particular the stimulation of cytotoxic CD8+ T cell responses. These results could inform better timing of CD4+ T cell measurements in future clinical trials.

## Introduction

More than thirty years after the identification of the human immunodeficiency virus (HIV) as the etiological agent of acquired immunodeficiency syndrome (AIDS), the global HIV epidemic remains one of the major global health challenges [1,2]. The development of a safe and efficacious prophylactic vaccine strategy against HIV constitutes an opportunity to control the pandemic.

Several clinical trials have been conducted in recent years to evaluate prophylactic strategies combining different candidate vaccines, but showed at best modest efficacy [3–8]. Previous trials have especially highlighted the importance of the vaccine regimen, in particular the concept of prime and boost strategy to optimize vaccine efficacy [3,9,10].

Although correlates of protection in HIV vaccine trials are not yet validated, both cellular and humoral immune responses are likely required to achieve sufficient protective vaccine efficacy against HIV acquisition. The assessments of these immune responses therefore play a major role in phase I-II HIV vaccine trials and in the decision to proceed to large scale efficacy trials.

One goal of vaccine-induced cellular responses is to stimulate CD8+ cytotoxic responses able to effectively fight against the virus and to control the infection. IL-2 producing CD4+ T cells play a role in the generation of this response, as they stimulate the differentiation of CD8+ cytotoxic effector cells and memory cells. They also help for the differentiation of B cells. [11,12]. However, little is known about the relationships between the different markers of immunologic response in HIV vaccine recipients, and little data are available on the dynamics of immunologic responses over time.

The time points at which different immunogenicity markers are measured in phase I and II HIV vaccine trials vary but are usually selected between two and four weeks after the final vaccine injection [13]. A better understanding of the dynamics of vaccine-induced immune responses would help facilitate a sampling strategy that can target optimal time points for each immunogenicity marker, to learn more about the vaccine's immunogenicity and increase the efficiency of clinical trials.

We hypothesized that certain immunogenicity markers which are measured early after vaccination could predict the subsequent immune responses of other markers: in particular, the dynamics of IFN-γ producing cytotoxic CD8+ T cells could be predicted by the early dynamics of IL-2 producing CD4+ helper T cells, which are known to stimulate the maturation of CD8+ T cells [14–16].

The main objective of this work was to determine the dynamics and correlations between different cellular immunogenicity markers (IFN-γ producing cytotoxic CD8+ T cells and IL-2 producing CD4+ helper T cells) during a prophylactic HIV vaccine strategy, using the example of a phase I trial of prime-boost vaccine strategies with rAd5 and DNA candidate vaccines. The secondary objective was to determine the correlations between cellular and humoral (Env-binding antibodies) immunogenicity markers.

## Methods

### Study population

This study was based on individual data from HVTN (HIV Vaccine Trials Network) 068 (http://www.clinicaltrials.gov; NCT00270218), a phase I placebo-controlled multicenter trial comparing two different prophylactic HIV vaccine strategies in healthy HIV-uninfected volunteers. The first group (rAd5-rAd5 group) received recombinant adenovirus type 5 (rAd5) vector vaccine as a prime (week 0) and a rAd5 boost (week 24). The second group (DNA-rAd5 group) received two DNA vaccine primes (weeks 0 and 4), and a rAd5 boost (week 24). The vaccines encoded HIV-1 Env glycoprotein (clades A/B/C) and clade B Gag/Pol fusion gene.

Sixty-six participants (aged 18–50 years and lacking detectable pre-existing Ad5-neutralizing antibodies) were randomized, with 30 active vaccine recipients and three placebo recipients in each of the two groups. The institutional review committee at each clinical site approved the protocol prior to study initiation, and all participants provided a written informed consent to participate in this study. Details of this trial have been previously reported [17].

**Fig 1:**
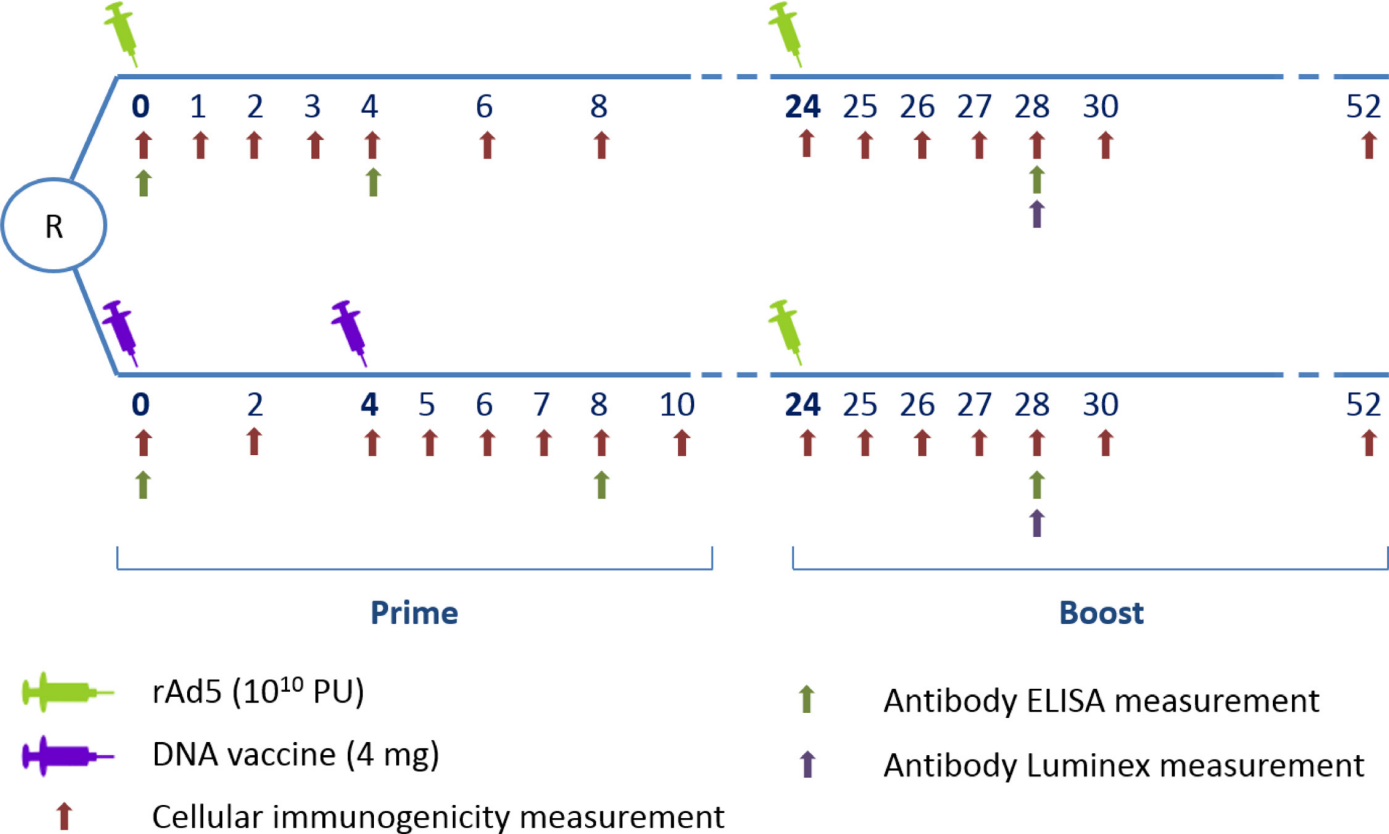
HVTN 068 trial design with repeated immunogenicity measurements–cellular and antibody responses—during the 52 weeks of follow-up.

The main interest in using HVTN 068 data for the present analysis was that early and repeated immunogenicity measurements were available following each vaccine injection (Fig 1). In the present analysis, only participants who received active vaccine were analyzed (60 participants).

### Immunogenicity measurements

Immunogenicity measurements were performed in batches in a central laboratory. At each cellular immunogenicity sampling time point (see Fig 1), a validated eight-color intracellular cytokine staining (ICS) assay [18] and a ten-color ICS assay were used to measure cytokine production following 6 hours stimulation of peripheral blood mononuclear cells with Env, Gag or Pol potential T cell epitope (PTE) peptide pools. After gating, percentages of CD4+ and CD8+ T-cells producing IL-2 or IFN-γ, respectively, were assessed using FlowJo (Treestar) or LabKey Flow software. The gating hierarchy is shown in Appendix A in S1 File. The present analyses focused on Env-specific responses because few Gag and Pol responses were detected [17].

Env binding antibody titers were measured using ELISA at three time points (at baseline, and four weeks after the prime and the boost vaccine injections) and using the multiplex bead array (Luminex) assay four weeks after the boost injection as previously described [17,19].

### Statistical analysis

All analyses were conducted separately for the DNA-rAd5 group and rAd5-rAd5 group, respectively. Associations between background-unsubtracted percentages of IFN-γ producing cytotoxic CD8+ T cells (CD8+ IFN-γ+) and IL-2 producing CD4+ (CD4+ IL-2+) helper T cells were assessed at each time point using Spearman correlations. We used background-unsubtracted responses for the main analysis of immune responses from HIV-uninfected individuals since with current knowledge it cannot be excluded that background responses may have some biological relevance [20]. Background-subtracted responses were analyzed in robustness analyses. Correlations between CD8+ IFN-γ+ T cells and CD4+ IL-2+ helper T cells and antibody responses (ELISA and Luminex) were also assessed. Multiplicity-adjusted p-values were calculated by using the Benjamini and Hochberg adaptive false discovery rate (FDR) method [21]. FDR-adjusted p-values < 0.10 were considered statistically significant.

Further modeling analyses were performed in vaccine groups with relevant correlations in the descriptive analyses. Modeling of the dynamics of the immune responses was performed using mixed effects regression models [22]. The mixed effects model assumptions were verified. The distribution of CD4+ IL-2+ and CD8+ IFN-γ+ were not entirely Gaussian but the use of these models is considered robust to moderate deviations from normality assumptions.

First, CD8+ IFN-γ+ T cells response was modeled as a function of time (expected visit day) using spline functions to fit the effect of follow-up time covering both prime- and boost injections. The set of values of time was divided into (k+1) intervals with nodes to define the limits of the intervals. In each interval, the relationship between the percentage of CD8+ IFN-γ+ T cell and time was modeled by a degree three polynomial [23,24]. Random intercepts and random slopes on each spline were used with an unstructured covariance matrix to take into account inter-participant variability.

Then, CD8+ IFN-γ+ T cells response was modeled as a function of both time and CD4+ IL-2+ helper T cell response in order to take into account the correlation between these two markers and to study more precisely the role of CD4+ IL-2+ helper T cells response in CD8+ IFN-γ+ response. The effect of time on the CD8+ IFN-γ+ response was included in this model using the spline functions previously defined.

Several models were compared to identify the one with the best fit using the Akaike information criterion (AIC) (smaller is better):

The first model (model 1 in Appendix B in S1 File) included the CD4+ IL-2+ response at each time as explanatory variables, and was adjusted for time using cubic B-splines.

In model 2 (model 2 in Appendix B in S1 File), CD4+ IL-2+ response was introduced as two explanatory variables: one taking values of observed CD4+ IL-2+ before week 2 (from baseline to week 1), and the second taking two values: 0 before week 2, and then the value observed at week 2 at each visit until the end of the follow-up. The first variable allowed for taking into account the CD4+ IL-2+ response before week 2, and the second variable the specific CD4+ IL-2+ response at week 2 on all following CD8+ IFN-γ+ measures. This choice, guided by the results of the descriptive correlation analyses, was made to more precisely study the association of the specific measure of CD4 + IL-2+ at week 2 on the entire subsequent CD8+ IFN-γ+ responses.

An additional exploratory analysis was performed to evaluate the robustness of the previous model using imputed CD4+ IL-2+ responses at theoretical time points around week 2 (at day 10 and day 17, respectively) obtained by linear interpolation.

All statistical analyses were done on available data using SAS (SAS Institute, Inc., Cary, NC) version 9.3 and R version 3.2.1.

## Results

**Fig 2:**
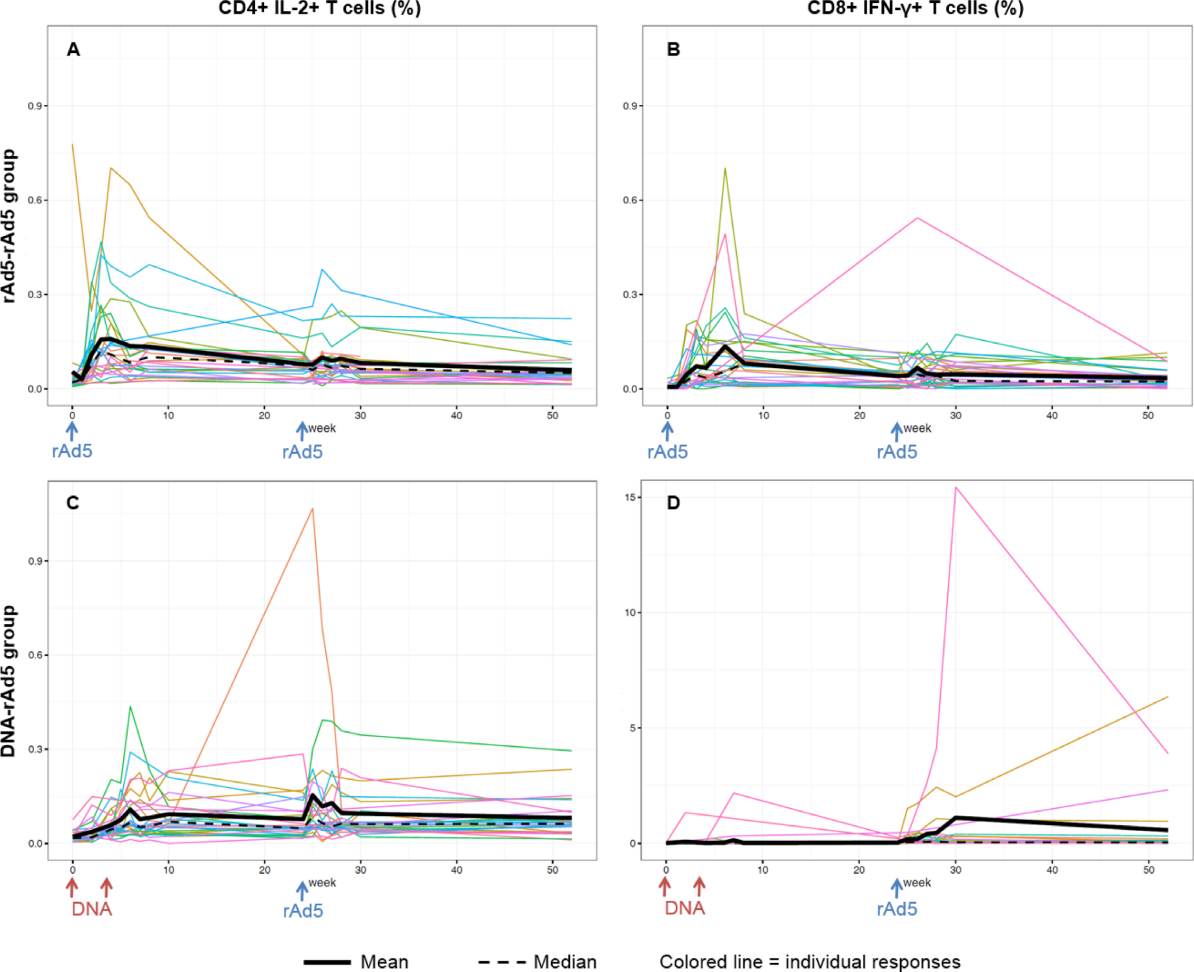
Kinetics of IL-2 producing CD4+ helper T cells (CD4+ IL-2+ T cells) and IFN-γ producing cytotoxic CD8+ T (CD8+ IFN-γ+ T cells) responses over time after Env ex-vivo stimulation of PBMC in the rAd5-rAd5 and DNA-rAd5 groups respectively, HVTN 068 trial. (A) CD4+ IL-2+ T cells responses in the rAd5-rAd5 group. (B) CD8+ IFN-γ+ T cells responses in the rAd5-rAd5 group. (C) CD4+ IL-2+ T cells responses in the DNA-rAd5 group. (D) CD8+ IFN-γ+ T cells responses in the DNA-rAd5 group.

Of the 30 participants randomized to each regimen, 26 rAd5-rAd5 participants and 28 DNA-rAd5 participants completed all vaccinations. The kinetics of their cellular immune responses—CD4+ IL-2+ T cells and CD8+ IFN-γ+ T cells, respectively—after Env peptide stimulation are shown in Fig 2.

**Fig 3:**
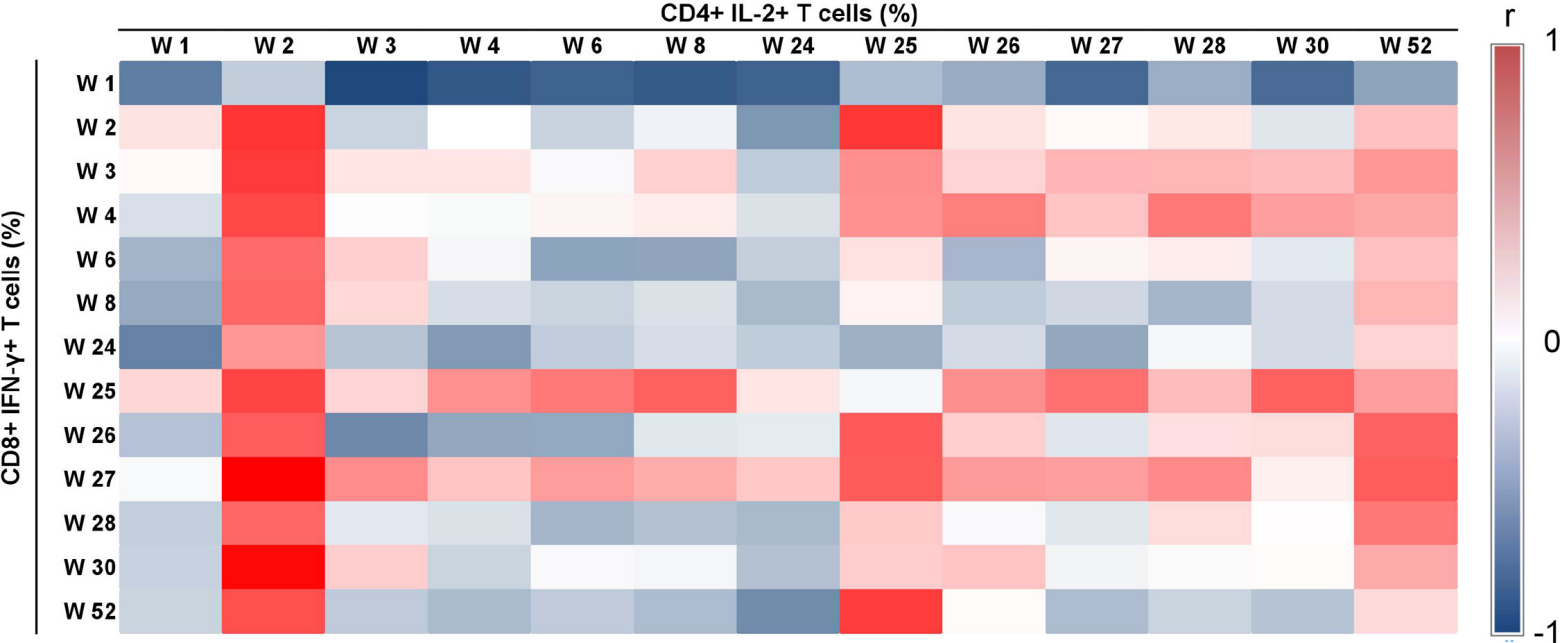
Heat map of Spearman correlation coefficients (r) between IL-2 producing CD4+ helper T cells (CD4+ IL-2+ T cells) and IFN-γ producing cytotoxic CD8+ T cells (CD8+ IFN-γ+ T cells) in the rAd5-rAd5 group (Env ex-vivo stimulation of PBMC), HVTN 068 trial.

A heat map of the Spearman correlations between CD4+ IL-2+ T cell and CD8+ IFN-γ+ T cell responses between all time points in the rAd5-rAd5 group is presented in Fig 3. In this group, moderate to high correlations (Spearman correlation coefficients ranging from 0.48 to 0.76) were observed between the percentage of CD4+ IL-2+ T cells at week 2 and the various measures of CD8+ IFN-γ+ T cells from week 2 until the end of the trial (Appendix C in S1 File). In contrast, no relevant correlations between CD4+ IL-2+ and CD8+ IFN-γ+ responses were found in the DNA-rAd5 group (Appendixes D and E in S1 File), and no significant correlations were found between CD4+ IL-2+ and antibody responses in either of the two groups. Robustness analyses using background-subtracted responses showed concordant results.

A complementary analysis of the correlation between CD4+ IL-2+ T cell and CD8+ IFN-γ+ T cell responses in the rAd5-rAd5 group using pooled Env, Gag and Pol peptides stimulation is presented in Appendix F in S1 File. Although the numbers of CD4+IL2+ at week 2 are still correlated (>0.5) with the CD8 response post-boost, the message is less clear than for Env only, as there are higher correlations between post boost CD4 and CD8 responses.

Guided by the results of the descriptive correlation analyses, regression modeling was performed only in the rAd5-rAd5 group using Env peptide stimulation data. The first model (model 1) fitted CD8+ IFN-γ as a function of CD4+ IL-2+ response at the same time point, and an additional effect of time using splines. In this model, CD4+ IL-2+ response was not significantly associated (p = 0.28) with CD8+ IFN-γ response (Table 1).

**Table 1:** Modeling of IFN-γ producing cytotoxic CD8+ T cells (CD8+ IFN-γ+ T cells) as a function of IL-2 producing CD4+ helper T cells (CD4+ IL-2+ T cells) and time (model 1 and model 2) in the rAd5-rAd5 group, HVTN 068 trial.

Models	CD4+ IL2+ Tcell (each time)		CD4+ IL2+ Tcell (day 0—day 7)		CD4+ IL2+ Tcell (day 14)		Time (splines)	AIC
	β	p-value	β	p-value	β	p-value	p-value	
Model 1	0.004	0.28	-	-	-	-	<0.01	-846.2
Model 2	-	-	-0.001	0.82	0.031	<0.01	<0.01	-860.7

Model 1: % of CD8+ IFN- modelled as a function of % of CD4+ IL-2+ response at each time, adjusted for time (splines).

Model 2: % of CD8+ IFN- modelled as a function of % of CD4+ IL-2+ response introduced as two explanatory variables (one taking values of observed % of CD4+ IL-2+ before week 2 and the second taking two values: 0 before week 2, and then the value observed at week 2 at each visit until the end of the follow-up), adjusted for time (splines)

The shown regression coefficients (β) correspond to the estimated increase in % of CD8+ IFN-γ+ T cells per 0.1% increase in CD4+ IL-2+. For an increase of 0.10% in the percentage of CD4+ IL-2+ at week 2, the percentage of CD8+ IFN-γ increased of 0.03% throughout follow-up (p<0.01). In contrast, the values of CD4+ IL-2+ before week 2 were not significantly associated with CD8+ IFN-γ+ response (p = 0.82).

Model 2 was the best model with the lowest (i.e. best) AIC (Table 1). In this model, CD4+ IL-2+ responses observed at week 2 post-prime were significantly associated with CD8+ IFN-γ+ response at all later time points, including time points after the boost: for an increase of 0.10% in the percentage of CD4+ IL-2+ at week 2, the percentage of CD8+ IFN-γ increased an average of 0.03% throughout follow-up (p<0.01). In contrast, the values of CD4+ IL-2+ before week 2 were not significantly associated with CD8+ IFN-γ+ response (p = 0.82).

In additional exploratory models with imputed CD4+ IL-2+ responses at day 10 and day 17 (obtained by linear interpolation, instead of using the week 2 measurement), CD4+ IL-2+ T cells were also significantly associated with CD8+ IFN-γ+ response (p<0.01), but had higher AICs.

## Discussion

We found a significant association in the rAd5-rAd5 group between the measurement of CD4+ IL-2+ responses 2 weeks following the prime and subsequent CD8+ IFN-γ+ T cell responses, including responses after the boost. This result emphasizes the role of early CD4+ helper cells in stimulating the response of CD8+ cytotoxic T cells [11,12]. IL-2 signals from CD4+ T cells affect CD8+ T cells during all stages of an immune response, including primary expansion, contraction, memory generation and secondary expansion [25–27]. One could hypothesize that the initial CD8+ T cell response may have little dependence on the CD4+ T cells during the first two weeks after prime vaccination, but that the production of IL-2 by antigen-stimulated CD4+ T cells intervenes to stimulate and sustain the CD8+ T cell response beyond that time.

The association with the measurement specifically at week 2 is novel in a HIV vaccine trial. First, this trial led to the opportunity to find this association because it featured early sampling times that are rarely performed. Similar trials typically have initial sample times that are a month or more after the prime, or even after the boost [28–31]. Second, we explored the effect of values of CD4+ T cell counts at several other time points based on the observed dynamics. Our finding of a specific association with a measure at week 2 indicates that adding this early sampling time point to future trials could be of interest at least for the measure of CD4+ T cell responses.

The main analyses focused on Env-specific responses because few Gag and Pol responses were detected [17]. However, we also looked at the correlation between the two cellular immunogenicity markers using pooled Env, Gag and Pol peptides stimulation. The message was less clear than for Env only, although the numbers of CD4+IL2+ at week 2 are still correlated (>0.5) with the CD8 response post-boost. This result could suggest a specific role of Env peptide stimulation in the early immune response post-prime compared to other peptides.

We found no significant correlations between cellular responses and antibody responses in this dataset. One might have expected a significant correlation because CD4+ helper T cells are also important for providing help to B cells in support of the humoral response [32]. Several hypotheses could explain the lack of significant correlations in our dataset. First, few sampling time points of serum antibody responses were available during follow-up, limiting the correlation analyses to a small number of time points. One cannot exclude the possibility that relevant correlation may exist for time points not observed in this trial. Second, these are measured markers in the blood and therefore may not represent the dynamics of immune response markers at other relevant tissue sites for vaccine-induced immune responses.

Immune responses to the vaccine strategies were different between the two arms of HVTN 068 trial, and no correlations between the CD4+ IL-2+ and CD8+ IFN-γ+ responses were observed in the group with the DNA vaccine prime. Indeed, few responses were detectable by intracellular cytokine staining following the DNA prime injections in this group, whereas responses to the boost were similar or higher than the ones in the rAd5-rAd5 group [17]. Previous studies have shown that DNA vaccines alone are not highly immunogenic in humans [33], but that they prime the responses for a heterologous boost. The immune effects elicited by a DNA prime may be too subtle to be detected by intracellular cytokine staining or may rely on a different immune mechanism than the ones usually assessed in clinical trials. In vitro expansion of CD4+ T cells could be relevant to evaluate the initial response in this type of situation but its association with the CD8 response remained to be demonstrated in this setting. Other immunological markers (e.g. transcriptome) may be helpful to better examine subtle effects of DNA vaccine primes [17]. Moreover, newer DNA vaccination strategies such as the process of electroporation, which facilitates the entrance of the DNA in cells, shows markedly better CD4+ and CD8+ vaccine responses [34]. These strategies might even be better for the modeling of DNA vaccines.

To our knowledge this is the first study modeling the immune response dynamics to a prophylactic HIV vaccine strategy in humans. Before generalizing our findings, validation in external datasets would be of interest. However, existing HIV vaccine clinical trial datasets with CD4+ cell response measurements early after the prime are scarce, and we have thus far not had the opportunity to perform an external validation of our hypothesis.

In conclusion, this study highlighted the early role of the CD4 helper T cell response, as measured by CD4+ IL-2+ T cells, in the cellular response to an Ad5-Ad5 vaccine strategy, and in particular in the stimulation of subsequent cytotoxic CD8 T cell responses. Early sampling time points should be considered in future clinical trials to better understand the role of the early CD4 helper T cells and to evaluate their predictive role in the immune response to vaccines.

## Supporting Information

S1 File (PDF)
