# Supplementary material for: Early CD4+ T Cell Responses Are Associated with Subsequent CD8+ T Cell Responses to an rAd5-Based Prophylactic Prime-Boost HIV Vaccine Strategy
Source: PLoS One. 2016 Apr 28;11(4):e0152952. doi: 10.1371/journal.pone.0152952 (PMC4849671; doi:10.1371/journal.pone.0152952)
Supplement: S1 File — (PDF) [file pone.0152952.s001.pdf]

Early CD4+ T cell responses are associated with subsequent CD8+ T cell responses to a rAd5-based prophylactic prime-boost HIV vaccine strategy

Edouard Lhomme, Laura Richert, Zoe Moodie, Chloé Pasin, Spyros A. Kalams, Cecilia Morgan, Steve Self, Stephen C. De Rosa, Rodolphe Thiébaut

Supporting Information

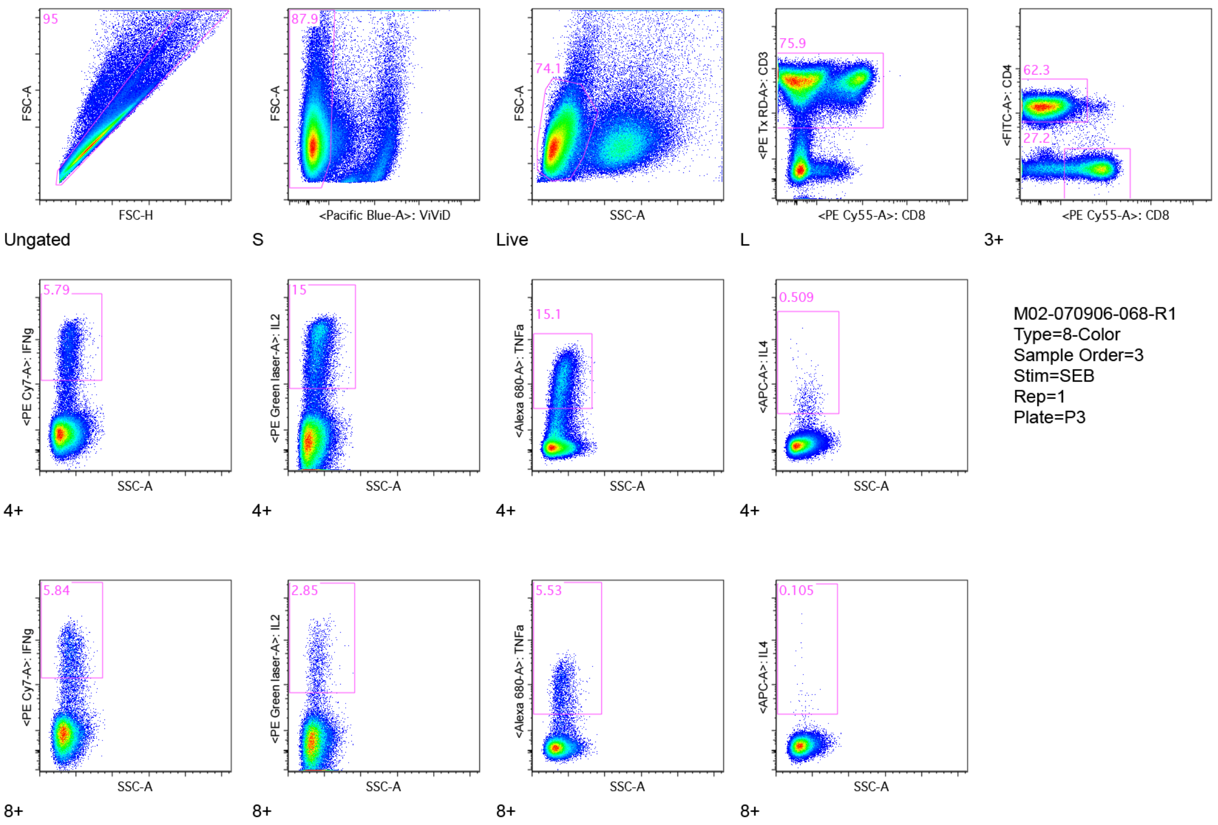

Appendix A. Example of gating hierarchy for 8-color ICS assay.

The upper row shows the series of gates to identify CD4+ and CD8+ T cells. In order, cells are gated for singlets, live cells, lymphocytes, CD3+ T cells and then CD4+ and CD8+ T cells. The middle and lower rows show the cytokine gates for CD4+ and CD8+ T cells, respectively. This is an example for the SEB positive control. The numbers in the gates show the percent of the gated cells of the parent gate.

Appendix B. Description of the two linear mixed regression models used to model IFN-γ producing cytotoxic CD8+ T cells (CD8+ IFN-γ+ T cells) as a function of IL-2 producing CD4+ helper T cells (CD4+ IL-2+ T cells) and time

Model 1

$$\text{LT CD8 IFN}\gamma_{ij} = \beta_0 + \beta_1 \text{ LT CD4 IL2}_{ij} + \sum_{k=1}^{K} \beta_k S_k(t) + \gamma_{0i} + \sum_{k=1}^{K} \gamma_k S_k(t) + \varepsilon_{ij}$$

where S_k are splines of time,

$\gamma_i = \begin{pmatrix} \gamma_{0i} \\ \gamma_{ki} \end{pmatrix} \sim N\left(\begin{bmatrix} 0 \\ 0 \end{bmatrix}, \begin{bmatrix} \sigma^2_{\gamma_0} & \sigma_{\gamma_0\gamma_k} \\ \sigma_{\gamma_0\gamma_k} & \sigma^2_{\gamma_k} \end{bmatrix}\right)$ and $\varepsilon_{ij} \sim N(0, \sigma^2_\varepsilon)$ are independent.

Model 2

$$\text{LT CD8 IFN}\gamma_{ij} = \beta_0 + \beta_1 \left(\text{LT CD4 IL2}_{ij}\right) I_{j<14} + \beta_2 (\text{LT CD4 IL2}_i)_{j=14}\, I'_{j\geq 14} + \sum_{k=1}^{K} \beta_k S_k(t)$$

$$+ \gamma_{0i} + \sum_{k=1}^{K} \gamma_k S_k(t) + \varepsilon_{ij}$$

where S_k are splines of time,

$\gamma_i = \begin{pmatrix} \gamma_{0i} \\ \gamma_{ki} \end{pmatrix} \sim N\left(\begin{bmatrix} 0 \\ 0 \end{bmatrix}, \begin{bmatrix} \sigma^2_{\gamma_0} & \sigma_{\gamma_0\gamma_k} \\ \sigma_{\gamma_0\gamma_k} & \sigma^2_{\gamma_k} \end{bmatrix}\right)$ and $\varepsilon_{ij} \sim N(0, \sigma^2_\varepsilon)$ are independent.

Appendix C. Spearman correlations between IFN-γ producing cytotoxic CD8+ T cells (CD8+ IFN-γ+ T cells) and IL-2 producing CD4+ helper T cells (CD4+ IL-2+ T cells) across follow-up in the rAd5-rAd5 group (Env ex-vivo stimulation of PBMC), HVTN 068 trial

CD8+ IFN-γ+ T cells (%)		CD4+ IL-2+ T cells (%)												
		Prime						Boost						
		**W 1**	**W 2**	**W 3**	**W 4**	**W 6**	**W 8**	**W24**	**W 25**	**W 26**	**W 27**	**W 28**	**W 30**	**W 52**
**Prime**														
**W 1**	r	-0.01	0.17	-0.14	-0.10	-0.08	-0.09	-0.08	0.14	0.10	-0.06	0.10	-0.06	0.07
	p-value	*0.89*	*0.63*	*0.69*	*0.73*	*0.74*	*0.73*	*0.74*	*0.72*	*0.73*	*0.79*	*0.73*	*0.79*	*0.75*
**W 2**	r	0.34	**0.67**	0.19	0.28	0.18	0.25	0.04	0.66	0.33	0.30	0.33	0.23	0.40
	p-value	*0.39*	***0.02***	*0.55*	*0.39*	*0.61*	*0.47*	*0.82*	***0.06***	*0.36*	*0.45*	*0.36*	*0.55*	*0.26*
**W 3**	r	0.30	**0.65**	0.33	0.33	0.27	0.37	0.16	0.50	0.36	0.42	0.42	0.41	0.48
	p-value	*0.45*	***0.02***	*0.33*	*0.33*	*0.47*	*0.35*	*0.64*	*0.26*	*0.33*	*0.33*	*0.26*	*0.33*	*0.18*
**W 4**	r	0.22	**0.63**	0.28	0.28	0.31	0.32	0.22	0.49	0.52	0.40	0.53	0.46	0.44
	p-value	*0.56*	***0.04***	*0.41*	*0.41*	*0.44*	*0.42*	*0.56*	*0.26*	*0.16*	*0.35*	*0.13*	*0.29*	*0.26*
**W 6**	r	0.11	0.56	0.37	0.27	0.07	0.07	0.17	0.34	0.12	0.30	0.32	0.24	0.40
	p-value	*0.71*	*0.18*	*0.38*	*0.48*	*0.74*	*0.74*	*0.63*	*0.44*	*0.70*	*0.44*	*0.43*	*0.50*	*0.33*
**W 8**	r	0.09	0.57	0.36	0.21	0.18	0.22	0.12	0.31	0.16	0.19	0.11	0.20	0.42
	p-value	*0.74*	*0.16*	*0.40*	*0.58*	*0.60*	*0.55*	*0.70*	*0.48*	*0.63*	*0.60*	*0.71*	*0.58*	*0.32*
**Boost**														
**W 24**	r	-0.00	0.48	0.15	0.05	0.17	0.21	0.16	0.10	0.20	0.08	0.27	0.20	0.37
	p-value	*0.92*	*0.26*	*0.68*	*0.81*	*0.63*	*0.58*	*0.63*	*0.74*	*0.58*	*0.74*	*0.48*	*0.58*	*0.39*

**W 25**	r	0.36	**0.63**	0.37	0.49	0.53	0.58	0.33	0.27	0.50	0.55	0.41	0.58	0.46
	p-value	*0.45*	***0.09***	*0.37*	*0.26*	*0.26*	*0.26*	*0.48*	*0.45*	*0.26*	*0.26*	*0.33*	*0.26*	*0.26*
**W 26**	r	0.15	**0.59**	0.01	0.08	0.08	0.23	0.24	0.59	0.37	0.23	0.34	0.35	0.58
	p-value	*0.67*	***0.10***	*0.89*	*0.74*	*0.74*	*0.55*	*0.53*	*0.16*	*0.32*	*0.58*	*0.36*	*0.39*	*0.11*
**W 27**	r	0.28	**0.76**	0.50	0.39	0.47	0.44	0.39	0.59	0.47	0.47	0.50	0.31	0.59
	p-value	*0.48*	***0.02***	*0.26*	*0.33*	*0.26*	*0.29*	*0.36*	*0.26*	*0.27*	*0.26*	*0.26*	*0.44*	*0.16*
**W 28**	r	0.18	**0.57**	0.23	0.21	0.11	0.14	0.12	0.38	0.27	0.23	0.35	0.29	0.54
	p-value	*0.61*	***0.10***	*0.26*	*0.50*	*0.71*	*0.67*	*0.70*	*0.33*	*0.44*	*0.56*	*0.33*	*0.46*	*0.14*
**W 30**	r	0.18	**0.75**	0.38	0.19	0.27	0.27	0.14	0.38	0.39	0.26	0.28	0.29	0.44
	p-value	*0.61*	***0.02***	*0.37*	*0.61*	*0.45*	*0.47*	*0.68*	*0.41*	*0.33*	*0.48*	*0.47*	*0.44*	*0.29*
**W 52**	r	0.19	**0.62**	0.16	0.13	0.16	0.13	0.01	0.65	0.29	0.13	0.18	0.14	0.35
	p-value	*0.61*	***0.05***	*0.60*	*0.66*	*0.63*	*0.68*	*0.88*	***0.06***	*0.42*	*0.69*	*0.58*	*0.67*	*0.29*

FDR-adjusted p-values < 0.10 were considered statistically significant and are presented in bold.

Appendix D.Spearman correlations between IFN-γ producing cytotoxic CD8+ T cells (CD8+ IFN-γ+ T cells) and of IL-2 producing CD4+ helper T cells (CD4+ IL-2+ T cells) across follow-up in the DNA-rAd5 group (Env ex-vivo stimulation of PBMC), HVTN 068 trial

CD8+ IFN-γ+ T cells (%)		CD4+ IL-2+ T cells (%)													
		Prime							Boost						
		W 2	W 4	W 5	W 6	W 7	W 8	W 10	W 24	W 25	W 26	W 27	W 28	W 30	W 52
**Prime**															
**W 2**	r	0.27	-0.38	-0.25	-0.22	0.04	0.00	0.14	-0.02	-0.25	-0.17	-0.21	0.03	0.02	-0.06
	p-value	*0.99*	*0.99*	*0.99*	*0.99*	*0.99*	*0.99*	*0.99*	*0.99*	*0.99*	*0.99*	*0.99*	*0.99*	*0.99*	*0.99*
**W 4**	r	0.02	-0.04	-0.07	-0.31	-0.3	-0.23	-0.13	-0.22	-0.26	-0.11	-0.33	-0.16	-0.17	-0.38
	p-value	*0.99*	*0.99*	*0.99*	*0.99*	*0.99*	*0.99*	*0.99*	*0.99*	*0.99*	*0.99*	*0.99*	*0.99*	*0.99*	*0.99*
**W 5**	r	0.04	-0.11	0.11	-0.23	-0.0	-0.10	-0.08	-0.19	-0.19	-0.03	-0.16	-0.02	-0.13	-0.19
	p-value	*0.99*	*0.99*	*0.99*	*0.99*	*0.99*	*0.99*	*0.99*	*0.99*	*0.99*	*0.99*	*0.99*	*0.99*	*0.99*	*0.99*
**W 6**	r	-0.38	0.12	-0.14	-0.22	0.18	0.01	-0.03	-0.09	-0.29	0.02	-0.21	0.13	-0.07	-0.27
	p-value	*0.99*	*0.99*	*0.99*	*0.99*	*0.99*	*0.99*	*0.99*	*0.99*	*0.99*	*0.99*	*0.99*	*0.99*	*0.99*	*0.99*
**W 7**	r	-0.04	0.17	0.50	0.19	0.11	0.20	0.33	0.13	0.05	-0.12	0.08	0.31	0.49	0.39
	p-value	*0.99*	*0.99*	*0.99*	*0.99*	*0.99*	*0.99*	*0.99*	*0.99*	*0.99*	*0.99*	*0.99*	*0.99*	*0.99*	*0.99*
**W 8**	r	-0.03	-0.18	0.02	-0.10	0.24	0.02	0.12	0.08	0.18	0.01	-0.10	0.16	0.18	0.06
	p-value	*0.99*	*0.99*	*0.99*	*0.99*	*0.99*	*0.99*	*0.99*	*0.99*	*0.99*	*0.99*	*0.99*	*0.99*	*0.99*	*0.99*
**W 10**	r	-0.15	-0.31	0.26	0.04	0.28	0.00	0.12	0.10	0.01	-0.02	-0.11	0.07	0.16	0.21
	p-value	*0.99*	*0.99*	*0.99*	*0.99*	*0.99*	*0.99*	*0.99*	*0.99*	*0.99*	*0.99*	*0.99*	*0.99*	*0.99*	*0.99*
**Boost**															
**W 24**	r	-0.13	-0.13	-0.02	-0.12	0.28	0.05	0.11	0.10	-0.09	-0.14	-0.29	0.15	0.03	-0.13

W 25	r	-0.05	0.20	0.24	-0.02	0.20	-0.16	-0.01	0.04	0.05	0.02	0.00	0.15	0.23	0.08
	p-value	*0.99*	*0.99*	*0.99*	*0.99*	*0.99*	*0.99*	*0.99*	*0.99*	*0.99*	*0.99*	*0.99*	*0.99*	*0.99*	*0.99*
W 26	r	-0.25	0.18	0.30	0.10	0.22	0.13	-0.07	-0.07	0.17	0.28	0.19	0.30	0.25	-0.04
	p-value	*0.99*	*0.99*	*0.99*	*0.99*	*0.99*	*0.99*	*0.99*	*0.99*	*0.99*	*0.99*	*0.99*	*0.99*	*0.99*	*0.99*
W 27	r	0.09	0.14	0.34	0.21	0.67	0.40	0.55	0.42	0.05	0.12	0.13	0.43	0.35	0.19
	p-value	*0.99*	*0.99*	*0.99*	*0.99*	*0.99*	*0.99*	*0.99*	*0.99*	*0.99*	*0.99*	*0.99*	*0.99*	*0.99*	*0.99*
W 28	r	-0.06	0.10	0.31	0.21	0.51	0.29	0.32	0.37	0.05	0.18	0.06	0.33	0.27	0.05
	p-value	*0.99*	*0.99*	*0.99*	*0.99*	*0.99*	*0.99*	*0.99*	*0.99*	*0.99*	*0.99*	*0.99*	*0.99*	*0.99*	*0.99*
W 30	r	-0.06	0.01	0.32	0.11	0.42	0.19	0.28	0.21	0.06	0.05	0.08	0.26	0.34	0.10
	p-value	*0.99*	*0.99*	*0.99*	*0.99*	*0.99*	*0.99*	*0.99*	*0.99*	*0.99*	*0.99*	*0.99*	*0.99*	*0.99*	*0.99*
W 52	r	-0.13	0.21	0.34	0.16	0.32	0.30	0.18	0.23	-0.01	0.12	0.11	0.24	0.20	0.14
	p-value	*0.99*	*0.99*	*0.99*	*0.99*	*0.99*	*0.99*	*0.99*	*0.99*	*0.99*	*0.99*	*0.99*	*0.99*	*0.99*	*0.99*

FDR-adjusted p-values < 0.10 were considered statistically significant and are presented in bold.

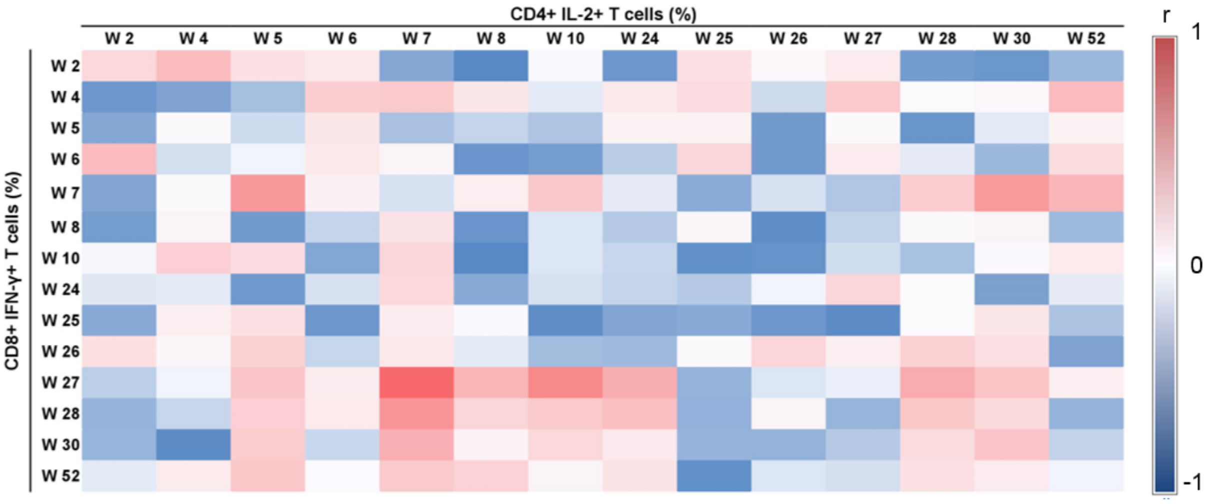

Appendix E. Heat map of Spearman correlation (r) between IL-2 producing CD4+ helper T cells (CD4+ IL-2+ T cells) and IFN-γ producing cytotoxic CD8+ T cells (CD8+ IFN-γ+ T cells) in the DNA-rAd5 group (Env ex-vivo stimulation of PBMC), HVTN 068 trial.

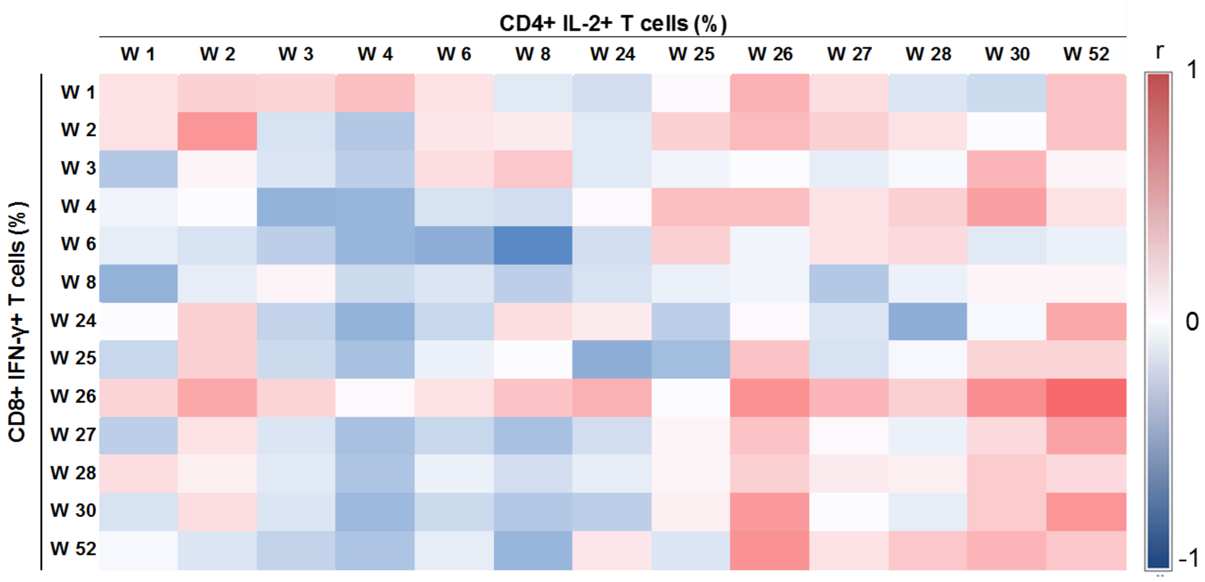

Appendix F. Heat map of Spearman correlation (r) between IL-2 producing CD4+ helper T cells (CD4+ IL-2+ T cells) and IFN-γ producing cytotoxic CD8+ T cells (CD8+ IFN-γ+ T cells) in the rAd5-rAd5 group (pooled Env, Gag and Pol ex-vivo stimulation of PBMC), HVTN 068 trial.
